# The Effect of Ginseng (The Genus *Panax*) on Glycemic Control: A Systematic Review and Meta-Analysis of Randomized Controlled Clinical Trials

**DOI:** 10.1371/journal.pone.0107391

**Published:** 2014-09-29

**Authors:** Esra' Shishtar, John L. Sievenpiper, Vladimir Djedovic, Adrian I. Cozma, Vanessa Ha, Viranda H. Jayalath, David J. A. Jenkins, Sonia Blanco Meija, Russell J. de Souza, Elena Jovanovski, Vladimir Vuksan

**Affiliations:** 1 Risk Factor Modification Centre, St. Michael's Hospital, Toronto, ON, Canada; 2 Department of Nutritional Sciences, Faculty of Medicine, University of Toronto, Toronto, ON, Canada; 3 Departments of Pathology and Molecular Medicine, Faculty of Health Sciences, McMaster University, Hamilton, ON, Canada; 4 Department of Clinical Epidemiology, Faculty of Health Sciences, McMaster University, Hamilton, ON, Canada; 5 Department of Medicine, Faculty of Medicine, University of Toronto, Toronto, ON, Canada; 6 Li Ka Shing Knowledge Institute, St. Michael's Hospital, Toronto, ON, Canada; Alberta Research Centre for Health Evidence, University of Alberta, Canada

## Abstract

**Importance:**

Despite the widespread use of ginseng in the management of diabetes, supporting evidence of its anti-hyperglycemic efficacy is limited, necessitating the need for evidence-based recommendations for the potential inclusion of ginseng in diabetes management.

**Objective:**

To elucidate the effect of ginseng on glycemic control in a systematic review and meta-analysis of randomized controlled trials in people with and without diabetes.

**Data sources:**

MEDLINE, EMBASE, CINAHL and the Cochrane Library (through July 3, 2013).

**Study selection:**

Randomized controlled trials ≥30 days assessing the glycemic effects of ginseng in people with and without diabetes.

**Data extraction:**

Relevant data were extracted by 2 independent reviewers. Discrepancies were resolved by consensus. The Heyland Methodological Quality Score and the Cochrane risk of bias tool were used to assess study quality and risk of bias respectively.

**Data synthesis:**

Sixteen trials were included, in which 16 fasting blood glucose (n = 770), 10 fasting plasma insulin (n = 349), 9 glycated hemoglobin (n = 264), and 7 homeostasis model assessment of insulin resistance (n = 305) comparisons were reported. Ginseng significantly reduced fasting blood glucose compared to control (MD =  −0.31 mmol/L [95% CI: −0.59 to −0.03], *P* = 0.03). Although there was no significant effect on fasting plasma insulin, glycated hemoglobin, or homeostasis model assessment of insulin resistance, *a priori* subgroup analyses did show significant reductions in glycated hemoglobin in parallel compared to crossover trials (MD = 0.22% [95%CI: 0.06 to 0.37], *P* = 0.01).

**Limitations:**

Most trials were of short duration (67% trials<12wks), and included participants with a relatively good glycemic control (median HbA1c non-diabetes = 5.4% [2 trials]; median HbA1c diabetes = 7.1% [7 trials]).

**Conclusions:**

Ginseng modestly yet significantly improved fasting blood glucose in people with and without diabetes. In order to address the uncertainty in our effect estimates and provide better assessments of ginseng's anti-diabetic efficacy, larger and longer randomized controlled trials using standardized ginseng preparations are warranted.

**Trial Registration:**

ClinicalTrials.gov NCT01841229

## Introduction

Diabetes is reaching epidemic proportions globally, with rates continually rising in both the developed and developing countries [1]. Despite advances in treatment, long-term diabetes management goals generally remain unmet [2]. Meanwhile, interest in complementary and alternative medicine (CAM) continues to grow [3], becoming one of the major therapeutic approaches sought by individuals with diabetes [4]. Among the most prevalent herbal CAMs, is American ginseng, which has demonstrated significant promise in the management of type 2 diabetes (T2DM), as acknowledged by the 2002 American Diabetes Association nutrition recommendations review [5].

Currently, 13 species of ginseng have been identified, of which Asian ginseng (*Panax ginseng)* and American ginseng (*Panax quinquefolius)*are the most extensively used and researched. Its pharmacological activity has been attributed to a group of saponins, also known as ginsenosides [6].

Several controlled clinical trials using American or Asian ginseng have demonstrated its therapeutic potential for glycemic control [7]–[9]. However, systematic reviews of such trials investigating the effect of ginseng on glycemic and metabolic parameters were largely inconclusive, concluding a lack of convincing evidence for benefit [10], [11] or reporting promising results for improving glucose metabolism [12], [13]. A previously conducted systematic review and meta-analysis on a single variety of ginseng, Korean red ginseng, did not show favorable outcomes in the management of T2DM, but was limited to 4 trials with incompatible study designs, as both acute and long-term effects of Korean red ginseng were investigated [14]. Therefore, our objective was to conduct a systematic review and meta-analysis of randomized controlled trials (RCTs) assessing for the first time, the glycemic effects of all species of ginseng (the genus *Panax*) in people with and without diabetes.

## Methods

Our meta-analysis followed the Cochrane Handbook for Systematic Reviews of Interventions [15]. We reported our findings according to the Preferred Reporting Items for Systematic reviews and Meta-Analyses (PRISMA) guidelines [16]. The review protocol is available online at ClinicalTrials.gov (registration number, NCT01841229).

### Data sources and searches

MEDLINE, EMBASE, CINAHL, and the Cochrane Central Register of RCTs were searched from inception through 3 July 2013, using a comprehensive search strategy (**Table S1**). Manual searches of the reference lists of all selected and review articles supplemented the electronic search. Abstracts and dissertations were also included with no restriction on language.

### Study selection

We included RCTs ≥ 30 days which examined the effect of oral ginseng supplementation (all species of the genus *Panax*) on at least one of four endpoints of glycemic control: fasting blood glucose (FBG), fasting plasma insulin (FPI), glycated hemoglobin (HbA1c), and homeostasis model assessment of insulin resistance (HOMA-IR), in individuals with and without diabetes. In order to isolate the effect of ginseng, trials that used ginseng as part of a multi-herbal treatment were excluded. Trials that lacked a suitable control, where ginseng was not adequately compared to either a placebo or a control group that was exposed to similar study conditions with the exception of not receiving the ginseng intervention, or those that did not provide suitable endpoints data were also excluded.

### Data extraction and quality assessment

Data were reviewed and extracted by two independent reviewers (E.S., V.D.) using a standardized *pro forma*. Relevant data extracted included information on authorship, publication year, study design, follow-up duration, blinding, sample size, subject characteristics, ginseng species, preparation, dose and form, comparator, and funding source. Study quality in included reports was assessed using the Heyland Methodological Quality Score (MQS), where a score ≥ 8 was considered to be high quality [17]. Risk of bias was assessed using the Cochrane Risk of Bias tool [15]. Discrepancies were resolved by consensus.

Mean ± SD for baseline values, change from baseline differences, end differences in FBG, FPI, HbA1c, and HOMA-IR were extracted. Missing SDs were imputed from 95% CI, *P* values, *t* or *F* statistics using published formulae [15]. When necessary, end-of-study changes in the treatment and control groups provided by individual trials were used to derive a correlation coefficient between intervention and control group outcomes. These correlations were then pooled using random effect modeling, where the pooled value was used to impute missing SDs [15], [18]. A calculated correlation (0.41) was used only for FPI imputation in paired analysis of crossover trials. Where needed, authors were contacted to request additional data.

### Data Synthesis and analysis

The mean difference (MD) and 95% CI was the summary outcome measure for all endpoints. Data for FBG, FPI, HbA1c, and HOMA-IR were aggregated using Review Manager (RevMan) software version 5.0.25 (The Nordic Cochrane Centre, The Cochrane Collaboration, Copenhagen, Denmark) for all primary analyses. Data were pooled using random effects models, with inverse variance weighting. Each endpoint was also stratified based on diabetes status. Between-treatment change from baseline differences were used as the primary end points and between-treatment end differences were used when these data were not available. All crossover trials underwent paired analyses [18], and the start value of the control was used when baseline values were not reported. In the event of multiple comparator arms within the same study, a weighted average was applied to create a single pairwise comparison [15]. Inter-study heterogeneity was assessed using the Cochran Q statistic with a significance level set at *P* <0.10, and quantified with the *I^2^* statistic where a value ≥ 50% reflected substantial heterogeneity. Potential sources of heterogeneity were investigated by *a priori* subgroup analyses of ginseng form, preparation, and species, follow-up duration, study quality and design, respective baseline glycemic parameters, and diabetes status. Further post-hoc subgroup analysis was undertaken to investigate possible effect modification by funding source. Meta-regressions were used to assess the significance of subgroup differences. The presence of a linear trend in the data based upon respective baseline glycemic parameters and follow-up duration were studied using continuous meta-regression analyses. Sensitivity analyses were performed to assess any undue influences of individual studies on the overall effect estimates, by systematically removing each individual study from the meta-analysis and recalculating the effect estimate from the remaining studies. Publication bias was examined through visual inspection of funnel plots and quantitatively evaluated by Begg's and Egger's tests. Meta-regressions and assessment of publication bias were performed on STATA 12 (StataCorp, College Station, Texas).

## Results

### Search results

**Figure 1:**
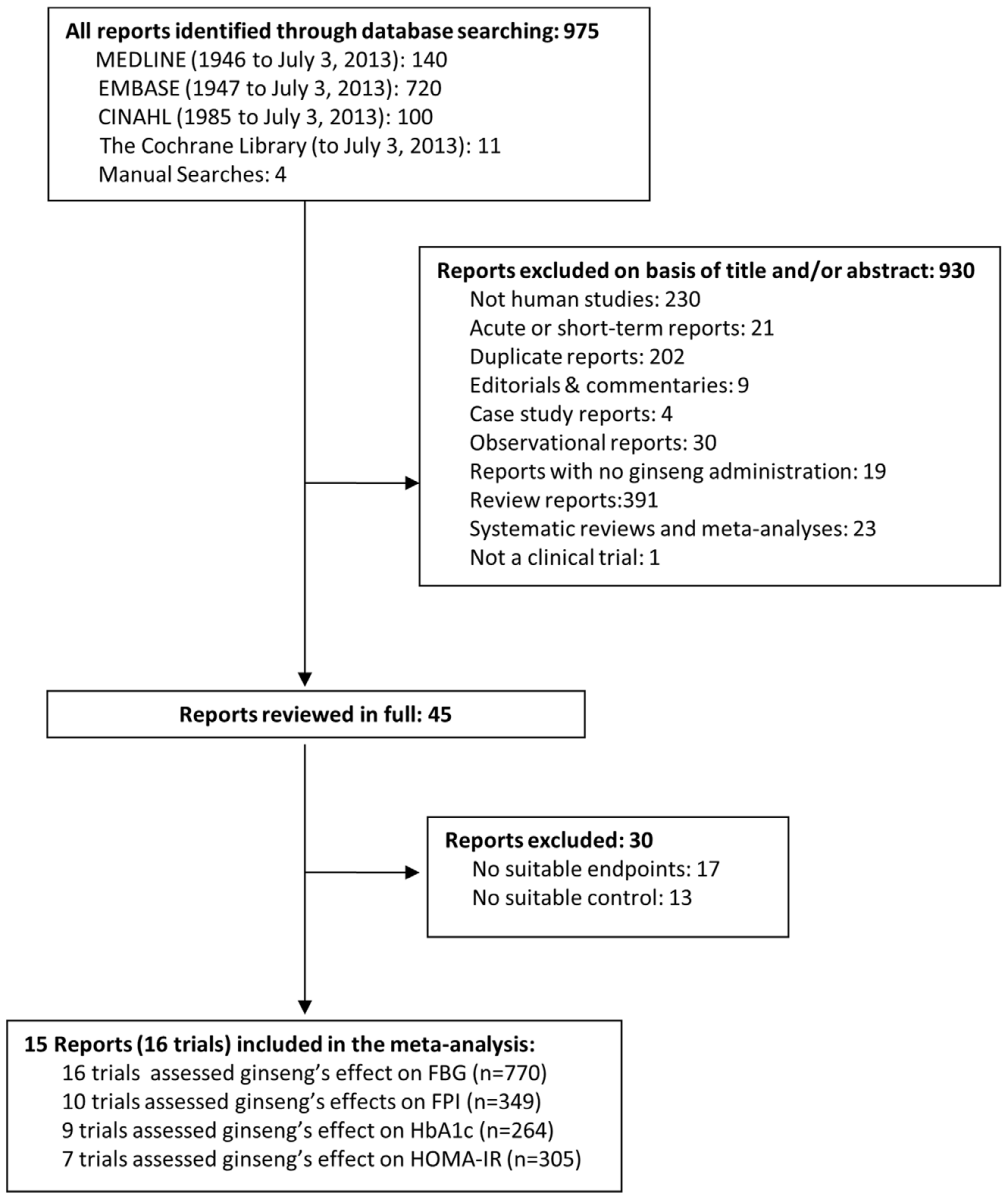
Flow of the literature search for the effect of ginseng on glycemic outcomes (FBG, FPI, HbA1c, and HOMA-IR).

**Figure 1** shows the flow of the literature. We identified 975 publications, of which 930 were excluded on the basis of title and abstract. Of the 45 potential relevant studies that were retrieved and fully reviewed, 30 were further excluded. Therefore, we included a total of 15 reports, providing data from 16 trials in 770 participants on the following endpoints: FBG (16 trial comparisons, n = 770), FPI (10 trial comparisons, n = 349), HbA1c (9 trial comparisons, n = 264), and HOMA-IR (7 trial comparisons, n = 305) [7], [9], [19]–[31].

### Trial characteristics

**Table 1** displays the characteristics of the included trials. Nine (56.3%) trials were conducted in 339 participants with diabetes and 7 (43.7%) in 431 participants without diabetes. Trials conducted on diabetes included subjects with type 1 diabetes mellitus (2 trials), type 2 diabetes mellitus (5 trials), and type 2 diabetes mellitus & pre-diabetes (2 trials). Non-diabetes trials included subjects with essential hypertension (1 trial), pre-diabetes (2 trials), and those that were otherwise healthy (4 trials). The median age of the study population was 51 years (IQR: 47.1–57.8 years). All but one trial [32] were carried out in outpatient settings. Fifty percent of all trials were conducted in Asia with the remaining conducted in North America and Europe (25% each). All trials assessed FBG in participants both with (9 trials; n = 339; median baseline FBG: 8.3 mmol/L (IQR: 7.8–8.8)) and without (7 trials; n = 431; median baseline FBG: 5.5 mmol/L (IQR: 5.1–5.8)) diabetes; ten trials assessed FPI in participants both with (7 trials; n = 272; median baseline FPI 70.8 pmol/L (IQR: 63.5–78.9)) and without (3 trials; n = 77; median baseline FPI 74.8 pmol/L (IQR: 74.4–86)) diabetes; nine trials assessed HbA1c in participants both with (7 trials; n = 235; median baseline HbA1c of 7.1% (IQR: 6.5–7.6) and without (2 trials; n = 29; median baseline HbA1c of 5.4% (IQR: 5.4–5.5)) diabetes; and seven trials assessed HOMA-IR in participants both with (6 trials; n = 257; median baseline HOMA-IR of 3.8 (IQR: 2.2–5.2)) and without (1 trial; n = 48; baseline HOMA-IR = 3) diabetes.

**Table 1:** Characteristics of Studies Investigating the Effect of Ginseng on Glycemic Outcomes.

Study*	Subject characteristics†	Age (y)	BMI (kg/m^2^)‡	Setting	Glucose (mmol/L)§	Insulin (pmol/L)§	HbA1c (%)§	HOMA-IR§	Design	Ginseng form	Ginseng preparation	Ginseng dose^∥^	Ginseng species	Comparator¶	Follow-up	MQS**	Funding Sources††	Manufacturer
Diabetes																		
Chio et al. 1997 [19]	31subjects with T1DM (15M:16F)	T: 51.6±10.9; C: 50.4±7.5	N/R	OP, Korea	T: 10.9±3.1; C: 13.2±4.4	N/R	T: 9.0±1.8; C: 10.1±2.6	N/R	Parallel	Capsule	Korean red ginseng (Unspecified preparation method)	2.7 g/d	*Panax ginseng*	No ginseng	24 wks	8	N/R	N/R
Kim et al. 2011 [21]	38 subjects with T2DM (23M:15F)	T: 56.0±7.2; C: 51.2±10.7	T: 24.8±2.7; C: 23.9±3.0	OP, Korea	T: 7.6±0.9; C: 8.1±1.5	T: 65.9±21.5; C: 63.9±20.8	T: 7.4±1.0; C: 7.5±1.2	T: 3.1±1.1; C: 3.2±1.2	Parallel	Capsule	Fermented Korean red ginseng (Unspecified preparation method)	780.0 mg/d	*Panax ginseng*	Placebo	12 wks	6	Agency	N/R
Ma et al. 2008 [22]	20 subjects with T2DM (12M:8F)	51.0± 8.5	28.5±5.8	OP, Hong Kong	8.3±3.1	149±111.9	N/R	7.4±3.8	Crossover	Capsule	Roots of *Panax ginseng*	2214.0 mg/d	*Panax ginseng*	Placebo	4 wks	8	Agency	N/R
Reeds et al. 2011 [25]	15 subjects with Pre-DM and T2DM (1M:14F)	46.0±11.6	T1: 35.0±6.7; T2: 36.0±4.5; C: 31.0±2.2	OP, USA	5.2±0.2	52.4±22.5	5.9±0.4	N/R	Parallel	Capsule	T1: Extract of Korean red ginseng; T2: Ginsenoside Re	T1: 3.0 g/d first 2wks, 8.0 g/d following 2wks; T2: 3.0 g/d first 2wks, 8.0 g/d following 2wks	*Panax ginseng*	Placebo	4 wks	10	Agency	Spectrum laboratories, Gardena AIPOP, Gangdown-Do, Korea
Sotaniemi et al. 1995 [7]	36 subjects with T1DM (16M:20F)	T1: 59.7±7.0; T2: 57±9.0; C: 60±6.0	N/R	OP, Finland	8.3±1.3	N/R	6.5±1.7	N/R	Parallel	Capsule	Extract of unspecified ginseng type	T1: 100.0 mg/d; T2: 200.0 mg/d	Unspecified	Placebo	8 wks	7	N/R	Dansk droge, Copenhagen, Denmark
Vuksan et al.2000 [29]	24 subjects with T2DM (13M:11F)	64.0±7.0	28.0±5.0	OP, Canada	8.3±2.5	88.1±66.9	7.1±0.1	5.4±3.9	Crossover	Capsule	Extract of American ginseng (CNT2000)	3.0 g/d	*Panax quinquefolius*	Placebo	8 wks	7	Industry	Chai-Na-Tai Corporation, Langley, BC, Canada
Vuksan et al. 2008 [9]	19 subjects with T2DM (11M:8F)	64.0±8.7	28.9±6.1	OP, Canada	7.7±1.7	35.0±17.4	6.5±0.3	1.7±0.9	Crossover	Capsule	Rootlets of Korean red ginseng	6.0 g/d	*Panax ginseng*	Placebo	12 wks	8	Agency	Korea Ginseng Manufacturing Plant, Chung-buk, Korea
Yoon et al. 2012 [30]	72 subjects with T2DM (44M:28F)	T1: 52.7±11.0; T2: 52.7±10.0; T3: 51.1±8.6; C: 54.8±10.0	T1: 26.3±4.8; T2: 24.0±2.6; T3: 25.4±2.7; C: 25.3±1.92	OP, Korea	T1: 9.2±2.2; T2: 9.6±1.9; T3: 8.9±1.5; C: 8.3±1.8	T1: 80.6±39.6; T2: 65.9±30.6; T3: 84.0±45.8; C: 68.8±29.2	T1: 7.8±1.3; T2: 7.8±1.2; T3: 7.7±0.8; C: 7.6±0.4	T1: 4.7±2.5; T2: 4.1±2.0; T3: 4.9±2.9; C: 3.8±2.1	Parallel	Capsule	Vinegar extract of *Panax ginseng* (Ginsam)	T1: 500.0 mg/d; T2: 2000.0 mg/d; T3: 3000.0 mg/d	*Panax ginseng*	Placebo	8 wks	10	Industry	YuYu Pharamaceutical Seoul, Korea
Zhang et al. 2007 [32]	84 subjects with Pre-DM and T2DM (51M:33F)	T: 62.9±12.0; C: 63.4±10.5	N/R	IP, China	7.7±1.91	58.8±57.4	N/R	1.1±0.57	Parallel	Capsule	Extract of *Panax quinquefolius* saponin (PQS)	1.8 g/d	*Panax quinquefolius*	No Ginseng	4 wks	8	Agency	Manufacturer name reported in Chinese
Non-diabetes																		
Dickman et al. 2009 [20]	25 Otherwise healthy females	T: 62.3±5.9; C: 61.7±6.5	T: 25.3±3.8; C: 24.7±2.5	OP, USA	4.6±0.5	N/R	N/R	N/R	Parallel	Capsule	Dry whole root of American ginseng	1.0 g/d	*Panax quinquefolius*	Placebo	16 wks	7	Agency	Kaiser Farms,Wausau,Wi
Park et al. 2012 [23]	48 Females with Pre-DM	T: 43.1±10.6; C: 46.2±11.0	N/R	OP, Korea	T: 5.9±1.0; C: 6.4±3.7	T: 77.0±60.4; C: 68.8±71.5	N/R	T: 3.1±2.9; C: 3.1±4.2	Parallel	Capsule	Roots of Korean red ginseng	4.5 g/d	*Panax ginseng*	Placebo	12 wks	8	Agency	Korea Ginseng Corporation, Seoul, Korea
Reay et al. (a) 2009 [24]	23 Otherwise healthy subjects (12M:11F)	35.6±1.1	N/R	OP, UK	4.9±0.9	72.9±28.0	5.5±0.3	N/R	Crossover	Capsule	Extract of *Panax Ginseng* (G115)	200.0 mg/d	*Panax ginseng*	Placebo	∼8 wks	10	Industry	Pharmaton SA, Lugano, Switzerland
Reay et al. (b) 2009 [24]	14 Otherwise healthy subjects (5M:9F)	38.4±10.6	N/R	OP, UK	5.7±0.5	97.2±36.8	5.5±0.36	N/R	Crossover	Capsule	Extract of *Panax ginseng* (Cheong Kwan Jang)	200.0 mg/d	*Panax ginseng*	Placebo	∼8 wks	10	Industry	Korea Ginseng Corporation, Seoul, Korea
Rhee et al. 2011 [26]	64 subjects with EHPT (28M:36F)	T: 55.0±9.0; C: 58.0±6.0	T: 24.9; C: 24.7	OP, Korea	T: 5.7±0.6; C: 5.6±0.6	N/R	N/R	N/R	Parallel	Capsule	Extract of Korean red ginseng	3.0 g/d	*Panax ginseng*	Placebo	12 wks	7	Agency & Industry	Korea Ginseg Co, Daejeon, Korea
Scaglione et al. 1996 [27]	227 Otherwise healthy subjects (132M:95F)	T: 48.0±16.4; C: 48.5±16.5	T: 23.5±1.2; C: 23.4±1.2	OP, Italy	5.3±0.7	N/R	N/R	N/R	Parallel	Capsule	Extract of *Panax ginseng (*G115)	200.0 mg/d	*Panax ginseng*	Placebo	12 wks	8	N/R	Pharmaton SA, Lugano, Switzerland
Shin et al. 2011 [28]	30 subjects with Pre-DM (18M:12F)	T: 47.1±10.8; C: 45.9±10.5	T: 24.9±7.4; C: 22.6±9.3	OP, Korea	T: 6.7±0.9; C: 6.2±0.9	N/R	N/R	N/R	Parallel	Capsule	Extract of Korean red ginseng with cheonggukjang (fermented soybean)	20.0 g/d	*Panax ginseng*	Cheonggukjang (fermented soybean)	8 wks	10	Agency	Keimyung Foodex Co, Daegu, Korea

Abbreviations: T1DM – Type 1 Diabetes mellitus; T2DM – Type 2 Diabetes mellitus; Pre-DM – Pre-diabetes Mellitus; EHPT – Essential hypertension; F – Female; M – Male; BMI – Body mass index; C – Control group; T – Treatment group; T1 – Treatment group #1; T2 – Treatment group #2; T3 – Treatment group #3; IP – Inpatient; OP – Outpatient; MQS – Heyland Methodological Quality Score; N/R – Not reported.

*Studies by Sotaniemi et al. [7], Yoon et al. [30], and Reeds et al. [25] contained multiple comparisons, and to mitigate unit-of-analysis error, we combined groups to create a single pairwise comparison.

† Pre-DM included subjects with either Impaired Fasting Glucose or Impared Glucose Tolerance.

‡ Pre-study baseline BMI is listed. The study by Rhee et al. [26] did not report SD for the mean BMI of participants.

§ Pre-study baseline endpoints are listed. In studies were these values were not reported, the start value of control was assumed to be equivalent to baseline and was reported. Where start of control value was not given, of control value was assumed to be equivalent to baseline and was reported. Assumed values are reported in bold. The study by Reay et al. (a) & (b) [24] used n = 23 and n = 14 respectively for reporting data on fasting blood glucose, n = 17 and n = 12 respectively for reporting data on fasting plasma insulin, and n = 18 and n = 11 respectively for reporting data on HbA1c.

∥Ginseng dose is reported individually for trials with multiple treatment groups.

¶ All ginseng doses were compared to placebo, a control group that did not receive ginseng, or fermented soybean.

**Study quality was assessed by the Heyland Methodological Quality Score (MQS) and trials with a score ≥ 8 were considered to be of high quality.

†† Agency funding is that from government, university or not-for-profit health agency sources. None of the trialists declared conflicts of interest.

All data is expressed as mean ± SD.

Eleven trials (68.8%) used parallel and five (31.3%) used crossover designs. Only two species of ginseng were identified across all trials: *Panax ginseng* (12 trials; 75%) and *Panax quinquefolius* (3 trials; 18.8%); one study, Sotaniemi et al., did not specify ginseng source. Ginseng preparations included whole root/rootlets of Korean red, American, or *Panax* ginseng (4 trials; 25%), and extracts of Korean red, American, or *Panax* ginseng (10 trials; 62.5%). Two trials (12.5%) did not specify method of preparation [19], [21]. All the trials used encapsulated powder forms of ginseng as the intervention. Thirteen trials (81.2%) used a placebo as the comparator, 2 (12.5%) used a control group that did not receive ginseng, and 1 (6.25%) used fermented soybean. The median follow-up was 8 weeks (IQR: 8–12).

Eleven trials (68.8%) were found to be of high quality (MQS ≥ 8). The median MQS among the available trials was 8 (IQR: 7–10) (**Table S2**). Of those trials which received low scores, the elements contributing to the low scores were poor description of randomization and treatment protocol, nonconsecutive or poorly described patient selection, high drop-out rates, and the absence of an intention-to-treat analysis. The Cochrane Risk of Bias Tool showed that 9 trials (56.3%) had unclear risk of bias and 7 trials (43.7%) had low risk of bias for sequence generation. Six trials (37.5%) had unclear risk of bias and 10 trials (62.5%) had low risk of bias for allocation concealment. Two trials (12.5%) had unclear risk of bias, 12 trials (75%) had low risk of bias, and 2 trials (12.5%) had high risk of bias for blinding. Fourteen trials (87.5%) were scored with low risk of bias, and 2 trials (12.5%) with high risk of bias for incomplete outcome data. Ten trials (62.5%) scored unclear risk, and 6 (37.5%) scored low risk of bias for selective outcome reporting (**Figure S1**). Eight (50%) studies reported funding from agency sources, four (25%) reported industry support, one (6.3%) reported agency-industry support, and three (18.7%) did not specify.

### Fasting Blood Glucose

**Figure 2:**
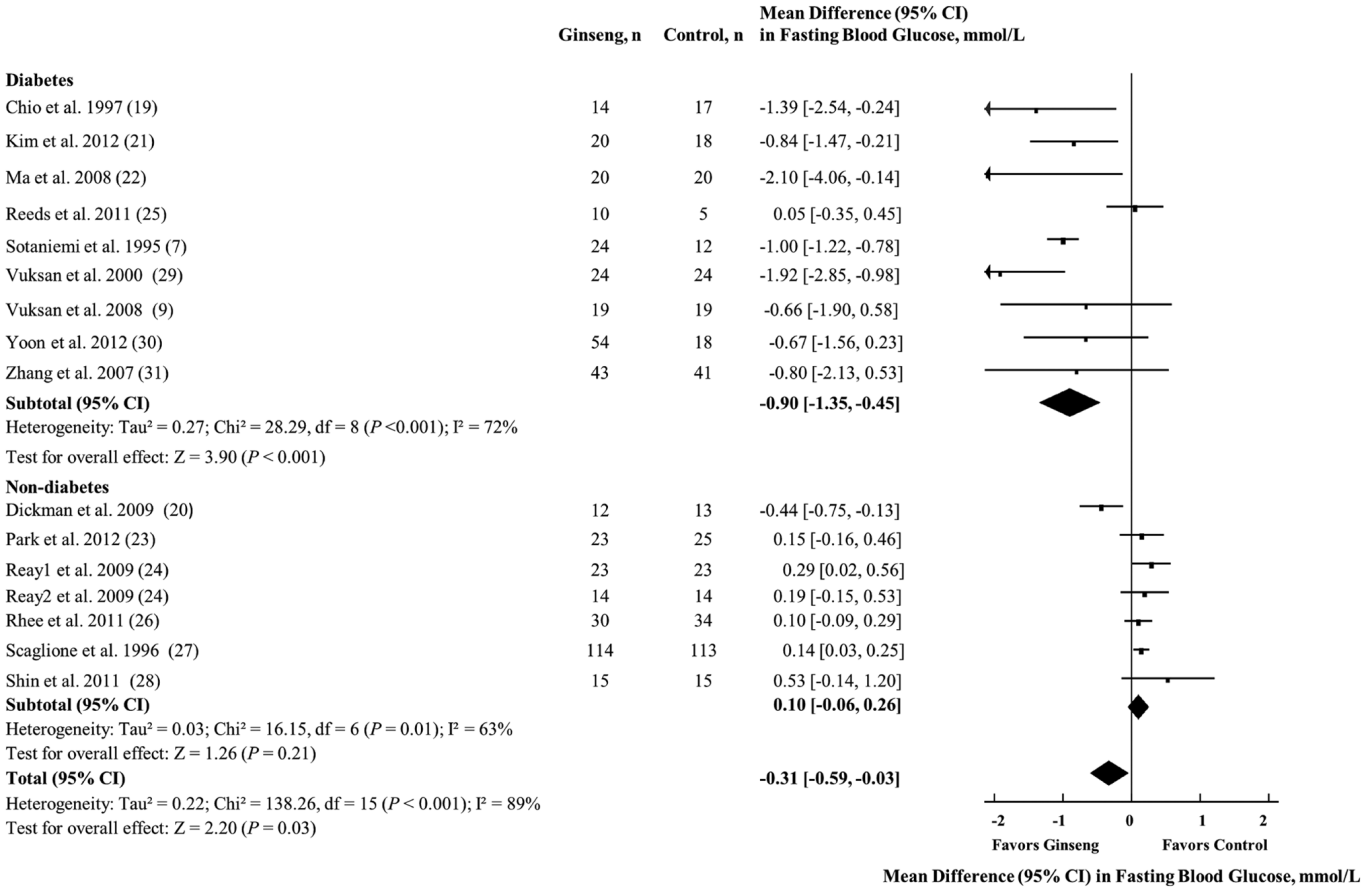
Forest plots of controlled clinical trials investigating the effect of ginseng on FBG. The diamond represents a pooled effect estimate. Paired analyses were applied to all crossover trials [18]. Data are mean differences (MD) with 95% CI. *P* values are for Generic Inverse Variance random effects models. Inter-study heterogeneity was tested by the Cochran Q statistic at a significance level of *P* <0.10 and quantified by the I^2^ statistic, where I^2^ ≥ 50% is considered to be evidence of substantial heterogeneity.

**Figure 2** shows the effect of ginseng supplementation on FBG. Overall, a significant FBG reduction was observed (MD = −0.31 mmol/L [95% CI: −0.59 to −0.03], *P* = 0.03). However, significant evidence of inter-study heterogeneity was seen in the overall analysis (I^2^ = 89%; *P* <0.001).

Sensitivity analyses of systematically removing individual trials showed that removal of five trials individually demonstrating benefits for FBG [7], [19], [21], [22], [29] led to a loss of significance in the overall effect (MD = −0.16 mmol/L; *P* = 0.14, MD = −0.27 mmol/L; *P* = 0.06, MD = −0.28 mmol/L; *P* = 0.06, MD = −0.28 mmol/L; *P* = 0.05, MD = −0.23 mmol/L; *P* = 0.1, respectively). *A priori* subgroup analyses revealed that the FBG-lowering effects of ginseng were only modified by the diabetes status of the individuals (between-group MD = −0.96 mmol/L [95% CI: −1.44 to −0.47], *P* = 0.001). Studies conducted in individuals with diabetes demonstrated a MD of −0.84 mmol/L [95% CI: −1.22 to −0.46], whereas those conducted in individuals without diabetes showed a MD of 0.11 mmol/L [95% CI: −0.19 to 0.42] (**Figure S2**). Continuous meta-regression analyses revealed a linear association between baseline FBG and treatment differences in FBG (β = −0.26 mmol/L [95% CI: −0.40 to −0.13] per 1 mmol/L, *P* = 0.001) (**Table S3**). Heterogeneity remained significant, and could not be explained away by any of the subgroup analyses.

### Fasting Plasma Insulin

**Figure 3:**
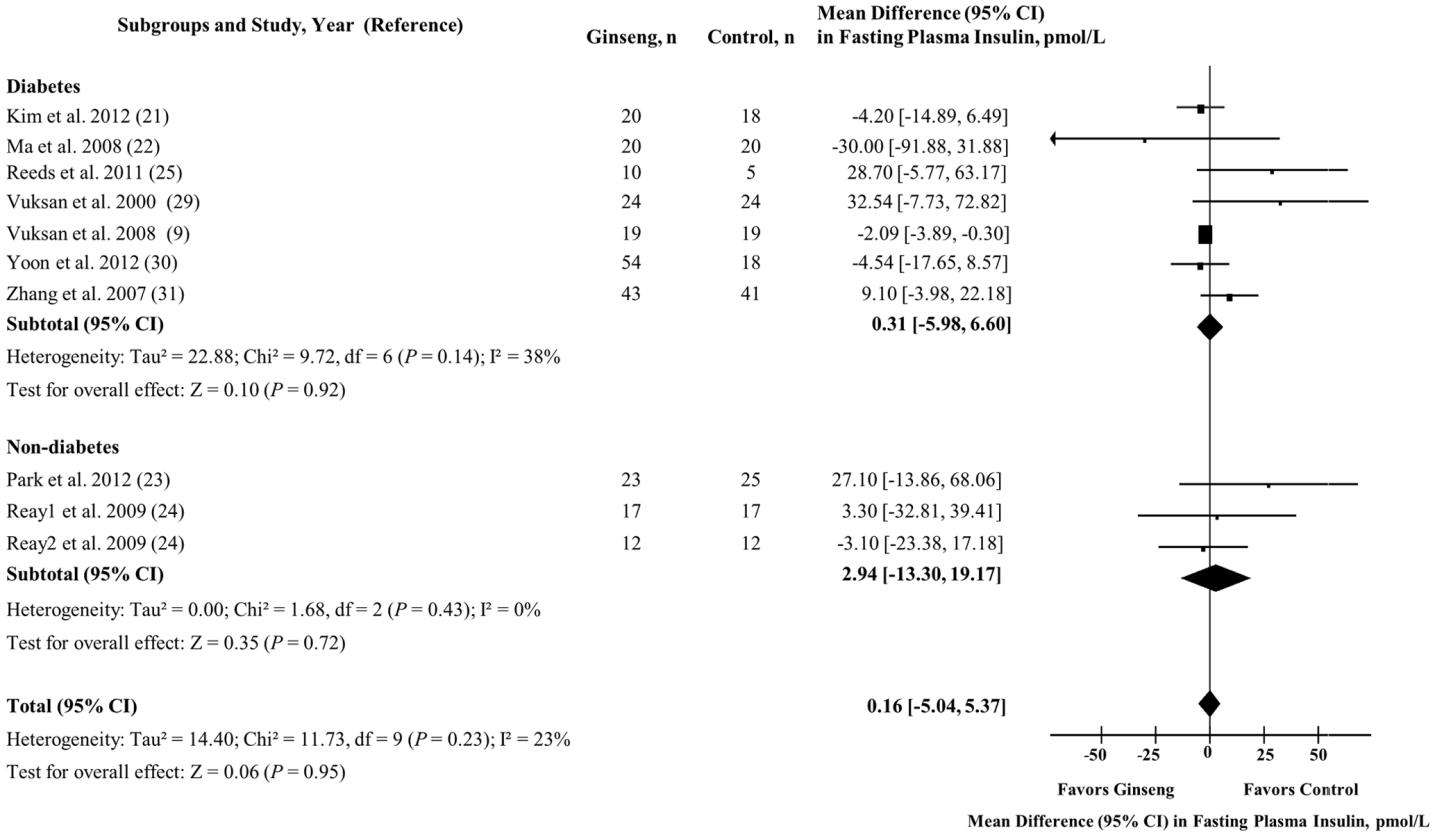
Forest plots of controlled clinical trials investigating the effect of ginseng on fasting plasma insulin. The diamond represents a pooled effect estimate. Paired analyses were applied to all crossover trials [18]. Data are mean differences (MD) with 95% CI. *P* values are for Generic Inverse Variance random effects models. Inter-study heterogeneity was tested by the Cochran Q statistic at a significance level of *P* <0.10 and quantified by the I^2^ statistic, where I^2^ ≥ 50% is considered to be evidence of substantial heterogeneity.

**Figure 3** shows the effect of ginseng supplementation on FPI. Overall, no significant difference in FPI levels were observed (MD = 0.16 pmol/L [95% CI: −5.04 to 5.37], *P* = 0.95), along with no evidence of inter-study heterogeneity (I^2^ = 23%; *P* = 0.23). No difference in effect estimate was identified by diabetes status.

Sensitivity analyses did not alter the direction or significance of effect estimates nor modify heterogeneity; and *a priori* subgroup analyses did not reveal significant effect modification by any subgroup under both dichotomous and continuous models (**Figure S3** and **Table S3**).

### Glycated Hemoglobin

**Figure 4:**
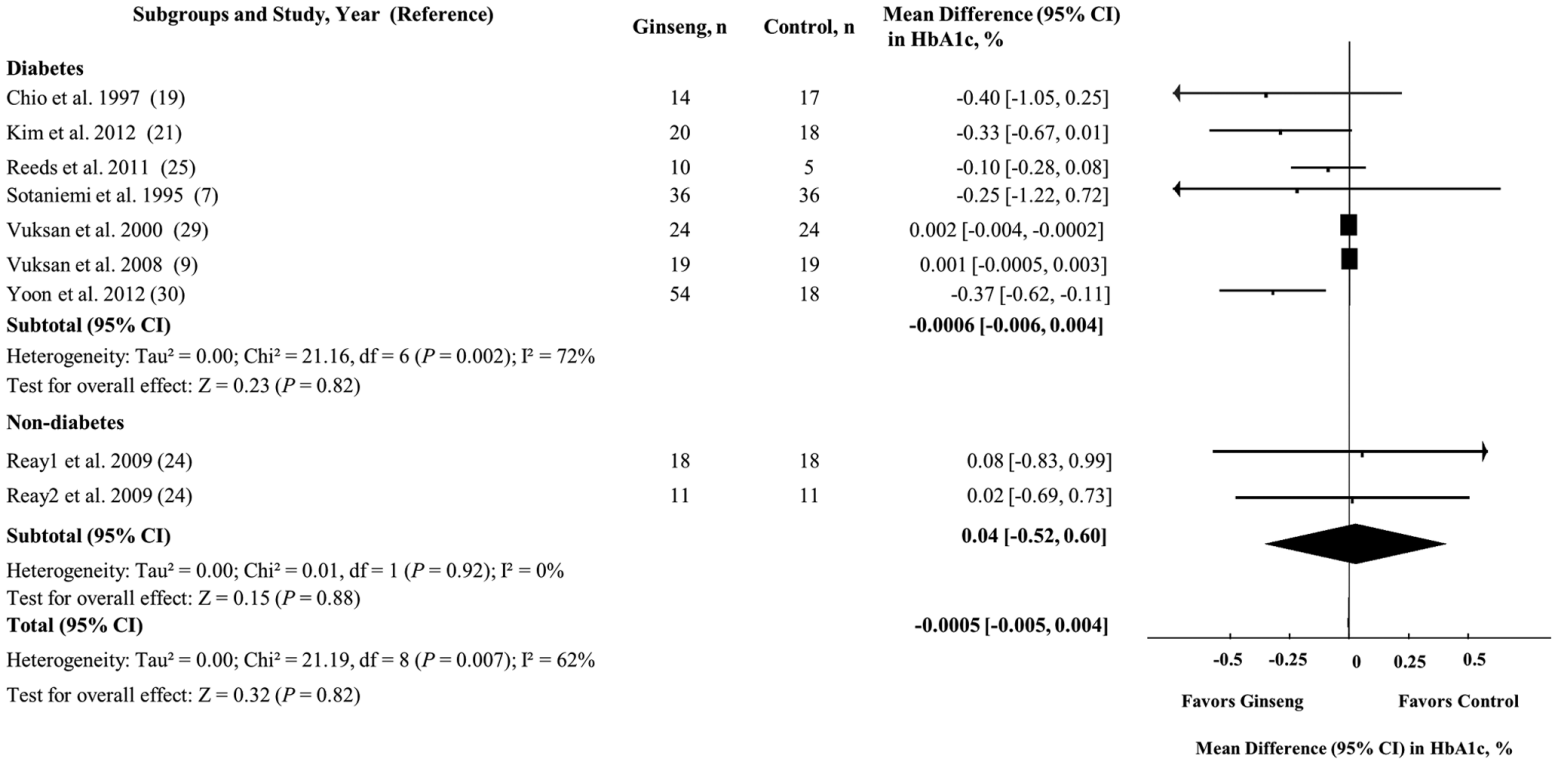
Forest plots of controlled clinical trials investigating the effect of ginseng on glycated hemoglobin. The diamond represents a pooled effect estimate. Paired analyses were applied to all crossover trial [18]. Data are mean differences (MD) with 95% CI. *P* values are for Generic Inverse Variance random effects models. Inter-study heterogeneity was tested by the Cochran Q statistic at a significance level of *P* <0.10 and quantified by the I^2^ statistic, where I^2^ ≥ 50% is considered to be evidence of substantial heterogeneity.

**Figure 4** shows the effect of ginseng supplementation on HbA1c. Ginseng supplementation did not change HbA1c levels in the overall analysis (MD = −0.0005% [95% CI: −0.005 to 0.004], *P* = 0.82), nor in the analyses by diabetes status. Nevertheless, significant inter-study heterogeneity was observed in the overall analysis (I^2^ = 62%, *P* = 0.007) and in the analyses in participants with diabetes (I^2^ = 72%, *P* = 0.002).

In our sensitivity analyses, the systematic removal of 2 individual trials [9], [29] resulted in a similar overall borderline significant effect, both demonstrating a MD of −0.15% [95% CI: −0.29 to −0.001], *P* = 0.05. The overall heterogeneity in the results was not affected by the individual removal of any studies. *A priori* subgroup analyses showed that reductions in HbA1c were only modified by study design (MD = 0.22% [95% CI: 0.06 to 0.37], *P* = 0.01). Crossover trials showed a MD of −0.0003% [95% CI: −0.004 to 0.003], whereas parallel trials demonstrated a MD of −0.22% [95% CI: −0.37 to −0.06] (**Figure S4**). No linear associations between baseline HbA1c or follow-up duration and reductions in HbA1c were found by continuous meta-regression analyses (**Table S3**).

### Homeostasis model assessment of insulin resistance

**Figure 5:**
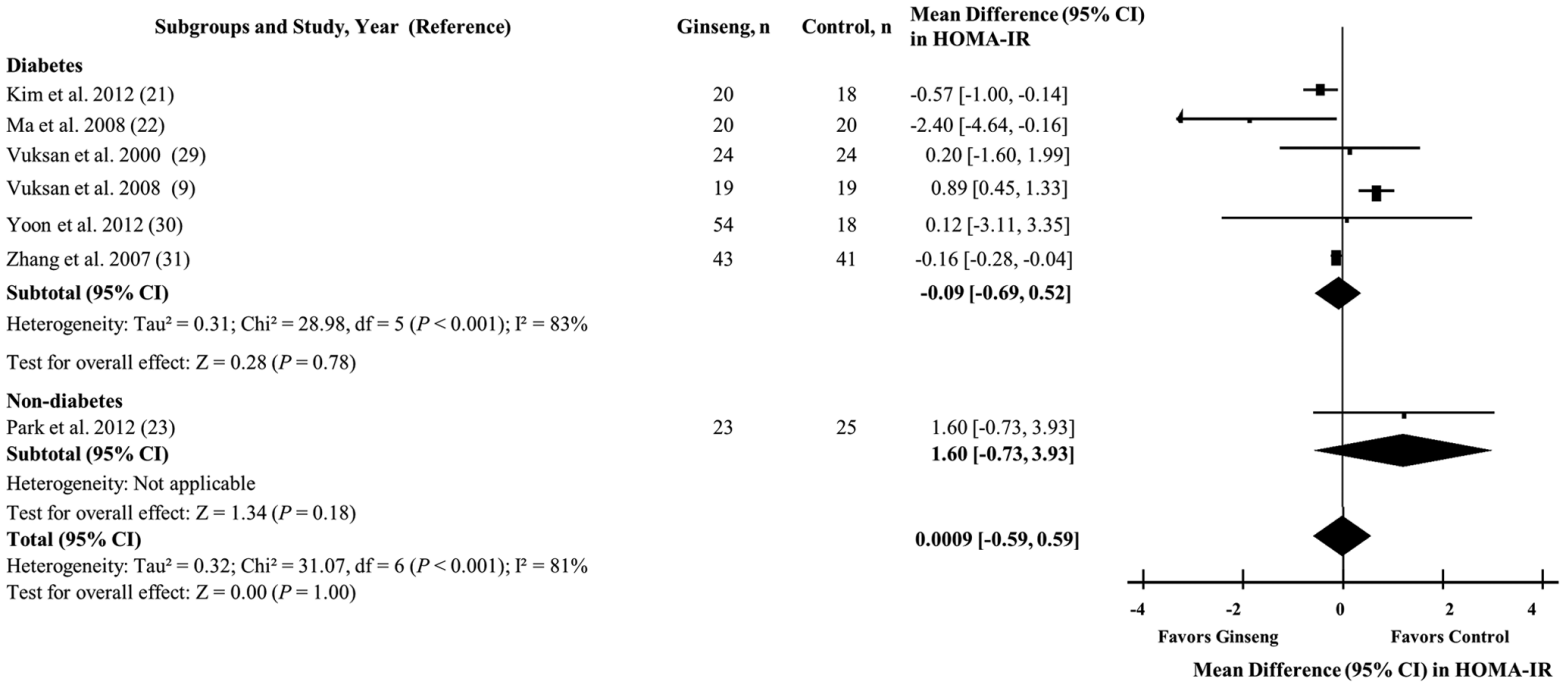
Forest plots of controlled clinical trials investigating the effect of ginseng on homeostasis model assessment of insulin resistance. The diamond represents a pooled effect estimate. Paired analyses were applied to all crossover trials [18]. Data are mean differences (MD) with 95% CI. *P* values are for Generic Inverse Variance random effects models. Inter-study heterogeneity was tested by the Cochran Q statistic at a significance level of *P* <0.10 and quantified by the I^2^ statistic, where I^2^ ≥ 50% is considered to be evidence of substantial heterogeneity.

**Figure 5** shows the effect of ginseng supplementation on HOMA-IR. Ginseng supplementation did not affect HOMA-IR levels in the overall analysis (MD = 0.0009 [95% CI: −0.59 to 0.59], *P* = 1.00), nor in analyses by diabetes. Although, significant inter-study heterogeneity was detected in the overall analysis (I^2^ = 81%, *P* <0.001).

Sensitivity analyses did not alter the direction or significance of effect estimates nor modify heterogeneity; and *a priori* subgroup analyses did not reveal significant effect modification by any subgroup under both dichotomous and continuous models. (**Figure S5** and **Table S3**).

### Publication Bias

Visual inspection of funnel plots suggested asymmetry in the FBG, FPI, and HbA1c analyses, with tendencies for the publication of small and/or imprecise trials favoring ginseng for FBG and HbA1c, and favoring comparator for FPI. However, this was not confirmed by either Egger's or Begg's tests (**Figure S6**).

## Discussion and Conclusions

An aggregate analyses of 16 RCTs in 770 participants showed that ginseng supplementation significantly lowered FBG. Ginseng intakes decreased FBG by 0.31 mmol/L (95% CI: −0.59 to −0.03, *P* = 0.03) following duration of ≥ 1month, in people with and without diabetes. Although there was no significant overall effect of ginseng on FPI, HbA1c, or HOMA-IR, *a priori* subgroup analyses did show a significant HbA1c benefit in parallel trials compared to crossover trials. Greater reductions in FBG were also observed in people with diabetes than those without diabetes.

Our findings add to those from previous systematic reviews in this area. Two recent systematic reviews assessing the efficacy and safety of ginseng reported promising, but inconclusive evidence for its application in moderating glucose metabolism [12], [13]. However, another earlier systematic review and meta-analysis failed to show a FBG-lowering effect of ginseng supplementation [14]. One reason for these inconsistencies may relate to differences in their eligibility criteria. Whereas the earlier systematic review included only RCTs that investigated the effect of Korean red ginseng in subjects with T2DM with either acute treatment administrations or treatment durations of least 12 weeks, we included trials which investigated the effect of any ginseng species in people with or without diabetes over at least 4 weeks. Despite having more inclusive criteria for ginseng species, diabetes status, and follow-up, we did not find any significant effect modification by any of these criteria with the exception of diabetes status.

Diabetes status partially explained the heterogeneity in the overall analysis for FBG. Participants with diabetes had a greater reduction in FBG than participants without diabetes. In line with this subgroup effect, our continuous meta-regression analyses showed that increases in baseline FBG were linearly associated with FBG reductions on the ginseng interventions, further supporting the notion that ginseng supplementation may generate a greater benefit in individuals with higher FBG levels.

It is unclear why the improvements in HbA1c were restricted to parallel trials only. One explanation may be an inadequate washout period between treatments in crossover designs, as it has been shown that ginseng metabolites may remain in the body for up to 10 weeks after treatment has ended [33]. As all of the crossover trial washouts were <10 weeks (range 27–42 days), confounding from carryover effects remains a strong possibility. Another explanation may relate to the glycemic control of the participants. It is a well understood phenomenon that the higher baseline HbA1c levels, the greater the fall with anti-hyperglycemic agents [34]. As most of the trials included participants with relatively good glycemic control in people with diabetes (median HbA1c 7.1%), it is possible that this resulted in blunted effects. The analysis also included people without diabetes (22% of trials) with optimal control (median HbA1c 5.4%) in whom a significant reduction in their HbA1c levels is unlikely to be observed [35]. Finally, as approximately 50% of the hemoglobin glycation reflected in HbA1c value occurs in 90–120 days [36], the short duration of the majority of included trials (6 of 9 trials that assessed HbA1c had <12 weeks follow-up) may have also underestimated the true HbA1c reductions.

The current results do not support the majority of evidence from animal and in-vitro data that suggest a potential of ginseng to increase insulin sensitivity and/or secretion [37]. Nevertheless, meta-regression by ginseng species found greater FPI reductions for *Panax ginseng* relative to *Panax quinquefolius*, although the between-subgroup difference was not significant (MD = −13.41pmol/L ([95% CI:−28.19 to 1.37], *P* = 0.07). These observations are line with findings from previous clinical trials supporting both the proposed insulin sensitizing and insulin secreting mechanisms for *Panax ginseng* and *Panax quinquefolius* respectively in the amelioration of hyperglycemia in T2DM [9], [29].

The mechanisms underlying ginseng's hypoglycemic activity remain unclear. A growing database of cell culture and animal studies indicate that ginseng may alleviate hyperglycemia by enhancing pancreatic β-cell function and reducing insulin resistance [38]. Data from these studies support four possible modes of anti-diabetic action: modulation of (1) glucose absorption, (2) insulin secretion and binding, (3) glucose transport, and/or (4) glucose disposal [37]. Collectively, these investigations offer preliminary but plausible explanations for the anti-diabetic potency of the two ginseng species in the clinical trials of this meta-analysis.

Assessment of the safety and tolerability of ginseng was not possible in this systematic review and meta-analysis, as only 4 of the 16 trials reported safety parameters among which a consistent safety parameter did not exist [9], [27], [29], [30]. Markers used in evaluating ginseng's safety included hepatic, renal, haemostatic, blood pressure function, comprehensive blood tests, and number of adverse events, none of which reported any difference in adverse events relative to the control. This parallels findings of several systematic reviews investigating the efficacy and safety of ginseng, where it was concluded that while its efficacy remains questionable, it appears to be generally safe [12], [37], [39]. Due to the nature of the intervention used in the trials, we could not eliminate the possibility of an effect modification by source of funding. Hence, following a post hoc analysis, no effect of funding source was found for any of the endpoints.

Several limitations of this systematic review and meta-analysis should be acknowledged. First, although undertaking dose-response analysis was specified *a priori*, it was not evaluated in the present work due to the variation in ginseng preparations administered (whole root/rootlets of Korean red/*Panax*/American ginseng vs. often uncharacterized extracts of these varieties), precluding calculation of ginseng dose equivalents. Second, the high variability in ginsenoside composition along with poor standardization of the ginsenoside profile continues to add complexity to the assessment of ginseng's glycemic benefits, as it has been shown that the anti-hyperglycemic efficacy of ginseng varies across species and is correlated to its ginsenoside composition [40]. To date, the optimal ratio of the most prominent bioactive components of ginseng, the ginsenosides, needed to ensure reproducible glucose lowering effects and product quality, has not been fully determined, necessitating a demand for better ginseng standardization. Third, information on the ginsenoside profile was not given by most of the trials, complicating the corroboration of a corresponding ginsenoside profile across studies that demonstrated effective findings. Finally, publication bias remains a concern as our funnel plot analyses demonstrated evidence of asymmetry favoring small and/or imprecise studies with FBG and HbA1c reducing effects, though this was neither confirmed by Egger's nor by Begg's test.

In conclusion, aggregate data analyses of controlled clinical trials show evidence for a modest yet significant benefit of ginseng in improving FBG in people with and without diabetes. Although ginseng did show advantages for HbA1c in parallel trials, the overall lack of an effect on HbA1c and persistent unexplained heterogeneity among the effect estimates from the available trials creates some uncertainty as to the long-term benefits of ginseng supplementation on glycemic control. The uncertainty points to several methodological limitations including the short duration of the trials, the well-controlled glycemia of participants at baseline, and the use of unstandardized ginseng preparations with potentially varying potencies. To provide more precise estimates of ginseng's long term effectiveness and address the unexplained heterogeneity, longer term, large scale, RCTs of the effect of ginseng preparations on HbA1c are warranted. Finally, given the promising effects of ginseng on FBG demonstrated herein, further research in this area is sensible, with a particular focus in investigating its potential glycemic-lowering components such as the unexamined non-saponins.

## Supporting Information

Figure S1 **Risk of bias assessment for included trials by the Cochrane risk of bias tool.**
(DOC)

Figure S2 **Forest plots of subgroup analyses investigating the effect of ginseng on fasting blood glucose.**
(DOCX)

Figure S3 **Forest plots of subgroup analyses investigating the effect of ginseng on fasting plasma insulin.**
(DOCX)

Figure S4 **Forest plots of subgroup analyses investigating the effect of ginseng on glycated hemoglobin.**
(DOCX)

Figure S5 **Forest plots of subgroup analyses investigating the effect of ginseng on homeostasis model assessment of insulin resistance.**
(DOCX)

Figure S6 **Funnel plot assessing publication bias and effect of small and/or imprecise study effects in clinical trials.**
(DOCX)

Table S1 **Search strategy for studies assessing the effect of ginseng on glycemic control in randomized controlled trials.**
(DOCX)

Table S2 **Study Quality Assessment by the Heyland MQS.**
(DOCX)

Table S3 **Continuous meta-regression analysis for the effect of ginseng on glycemic parameters.**
(DOCX)

Checklist S1 **PRISMA Checklist.**
(DOC)

## References

[1] King H, Aubert RE, Herman WH (1998) Global burden of diabetes, 1995–2025: prevalence, numerical estimates, and projections. Diabetes Care 21: 1414–1431. 9727886 10.2337/diacare.21.9.1414

[2] Levy P (2009) The current unmet need in type 2 diabetes mellitus: addressing glycemia and cardiovascular disease. Postgrad Med 121: 7–12. 10.3810/pgm.2009.05.suppl53.287 19494472

[3] Tackett KL, Jones MC (2009) Complementary and Alternative Medicines for the Treatment of Diabetes. Journal of Pharmacy Practice 22: 546–552.

[4] Chang HY, Wallis M, Tiralongo E (2007) Use of complementary and alternative medicine among people living with diabetes: literature review. J Adv Nurs 58: 307–319. 17442034 10.1111/j.1365-2648.2007.04291.x

[5] Franz MJ, Bantle JP, Beebe CA, Brunzell JD, Chiasson JL, et al (2002) Evidence-based nutrition principles and recommendations for the treatment and prevention of diabetes and related complications. Diabetes Care 25: 148–198. 11772915 10.2337/diacare.25.1.148

[6] Christensen LP (2009) Ginsenosides chemistry, biosynthesis, analysis, and potential health effects. Adv Food Nutr Res 55: 1–99. 18772102 10.1016/S1043-4526(08)00401-4

[7] Sotaniemi EA, Haapakoski E, Rautio A (1995) Ginseng therapy in non-insulin-dependent diabetic patients. Diabetes Care 18: 1373–1375. 8721940 10.2337/diacare.18.10.1373

[8] Tetsutani T, Amasaki K, Hima Y, No M (2000) Can red ginseng control blood glucose in diabetic patients? The Ginseng Review 28: 44–47.

[9] Vuksan V, Sung MK, Sievenpiper JL, Stavro PM, Jenkins AL, et al (2008) Korean red ginseng (Panax ginseng) improves glucose and insulin regulation in well-controlled, type 2 diabetes: results of a randomized, double-blind, placebo-controlled study of efficacy and safety. Nutr Metab Cardiovasc Dis 18: 46–56. 16860976 10.1016/j.numecd.2006.04.003

[10] Buettner C, Yeh GY, Phillips RS, Mittleman MA, Kaptchuk TJ (2006) Systematic review of the effects of ginseng on cardiovascular risk factors. Ann Pharmacother 40: 83–95. 16332943 10.1345/aph.1G216

[11] Vogler BK, Pittler MH, Ernst E (1999) The efficacy of ginseng. A systematic review of randomised clinical trials. Eur J Clin Pharmacol 55: 567–575. 10541774 10.1007/s002280050674

[12] Lee NH, Son CG (2011) Systematic review of randomized controlled trials evaluating the efficacy and safety of ginseng. J Acupunct Meridian Stud 4: 85–97. 21704950 10.1016/S2005-2901(11)60013-7

[13] Shergis JL, Zhang AL, Zhou W, Xue CC (2013) Panax ginseng in randomised controlled trials: a systematic review. Phytother Res 27: 949–965. 22969004 10.1002/ptr.4832

[14] Kim S, Shin BC, Lee MS, Lee H, Ernst E (2011) Red ginseng for type 2 diabetes mellitus: a systematic review of randomized controlled trials. Chin J Integr Med 17: 937–944. 22139546 10.1007/s11655-011-0937-2

[15] Higgins J, Green SE (2011) Cochrane handbook for systematic reviews of interventions version 5.1.0. The Cochrane Collaboration.

[16] Moher D, Liberati A, Tetzlaff J, Altman DG (2009) Preferred reporting items for systematic reviews and meta-analyses: the PRISMA statement. BMJ 339: b2535. 19622551 10.1136/bmj.b2535 PMC2714657

[17] Heyland DK, Novak F, Drover JW, Jain M, Su X, et al (2001) Should immunonutrition become routine in critically ill patients? A systematic review of the evidence. JAMA 286: 944–953. 11509059 10.1001/jama.286.8.944

[18] Elbourne DR, Altman DG, Higgins JP, Curtin F, Worthington HV, et al (2002) Meta-analyses involving cross-over trials: methodological issues. Int J Epidemiol 31: 140–149. 11914310 10.1093/ije/31.1.140

[19] Choi K, Lee E, Kim Y, Baik S, Kim Y, et al (1997) Effects of red ginseng on the lipid peroxidation of erythrocyte and antioxidant superoxide dismutase (SOD) activity in NIDDM patients. Korean J Ginseng Sci 21: 153–159.

[20] Dickman JR, Koenig RT, Ji LL (2009) American ginseng supplementation induces an oxidative stress in postmenopausal women. J Am Coll Nutr 28: 219–228. 19828907 10.1080/07315724.2009.10719773

[21] Kim HO, Park M, Han J (2011) Effects of fermented Red Ginseng supplementation on blood glucose and insulin resistance in type 2 diabetic patients. Journal of The Korean Society of Food Science and Nutrition 40: 696–703.

[22] Ma SW, Benzie IF, Chu TT, Fok BS, Tomlinson B, et al (2008) Effect of Panax ginseng supplementation on biomarkers of glucose tolerance, antioxidant status and oxidative stress in type 2 diabetic subjects: results of a placebo-controlled human intervention trial. Diabetes Obes Metab 10: 1125–1127. 18355331 10.1111/j.1463-1326.2008.00858.x

[23] Park BJ, Lee YJ, Lee HR, Jung DH, Na HY, et al (2012) Effects of Korean Red Ginseng on Cardiovascular Risks in Subjects with Metabolic Syndrome: a Double-blind Randomized Controlled Study. Korean J Fam Med 33: 190–196. 22916320 10.4082/kjfm.2012.33.4.190 PMC3418337

[24] Reay JL, Scholey AB, Milne A, Fenwick J, Kennedy DO (2009) Panax ginseng has no effect on indices of glucose regulation following acute or chronic ingestion in healthy volunteers. Br J Nutr 101: 1673–1678. 19017419 10.1017/S0007114508123418

[25] Reeds DN, Patterson BW, Okunade A, Holloszy JO, Polonsky KS, et al (2011) Ginseng and ginsenoside Re do not improve beta-cell function or insulin sensitivity in overweight and obese subjects with impaired glucose tolerance or diabetes. Diabetes Care 34: 1071–1076. 21411505 10.2337/dc10-2299 PMC3114517

[26] Rhee MY, Kim YS, Bae JH, Nah DY, Kim YK, et al (2011) Effect of Korean red ginseng on arterial stiffness in subjects with hypertension. J Altern Complement Med 17: 45–49. 21235416 10.1089/acm.2010.0065

[27] Scaglione F, Cattaneo G, Alessandria M, Cogo R (1996) Efficacy and safety of the standardised Ginseng extract G115 for potentiating vaccination against the influenza syndrome and protection against the common cold [corrected]. Drugs Exp Clin Res 22: 65–72. 8879982

[28] Shin SK, Kwon JH, Jeong YJ, Jeon SM, Choi JY, et al (2011) Supplementation of cheonggukjang and red ginseng cheonggukjang can improve plasma lipid profile and fasting blood glucose concentration in subjects with impaired fasting glucose. J Med Food 14: 108–113. 21128827 10.1089/jmf.2009.1366

[29] Vuksan V, Xu Z, Jenkins A, Beljan-Zdravkovic U, Sievenpiper J et al. (2000) American Ginseng improves long term glycemic control in type 2 diabetes: Double-blind placebo controlled crossover trial. American Diabetes Association Annual Meeting, Diabetes Suppl 1: 384.

[30] Yoon JW, Kang SM, Vassy JL, Shin H, Lee YH, et al (2012) Efficacy and safety of ginsam, a vinegar extract from Panax ginseng, in type 2 diabetic patients: Results of a double-blind, placebo-controlled study. J Diabetes Investig 3: 309–317. 10.1111/j.2040-1124.2011.00185.x PMC4014955 24843582

[31] Zhang Y, Lu S, Liu YY (2007) [Effect of panax quinquefolius saponin on insulin sensitivity in patients of coronary heart disease with blood glucose abnormality]. Zhongguo Zhong Xi Yi Jie He Za Zhi 27: 1066–1069. 18198636

[32] Zhang Y, Lu S, Liu YY (2007) Effect of panax quinquefolius saponin on insulin sensitivity in patients of coronary heart disease with blood glucose abnormality. Zhongguo Zhong Xi Yi Jie He Za Zhi 27: 1066–1069. 18198636

[33] Biondo PD, Robbins SJ, Walsh JD, McCargar LJ, Harber VJ, et al (2008) A randomized controlled crossover trial of the effect of ginseng consumption on the immune response to moderate exercise in healthy sedentary men. Appl Physiol Nutr Metab 33: 966–975. 18923572 10.1139/H08-080

[34] Canadian Diabetes Association Clinical Practice Guidelines Expert Committee (2013) Canadian Diabetes Association 2013 clinical practice guidelines for the prevention and management of diabetes in Canada. Can J Diabetes 37: S1–S212. 24070926 10.1016/j.jcjd.2013.01.009

[35] Simon D, Senan C, Garnier P, Saint-Paul M, Papoz L (1989) Epidemiological features of glycated haemoglobin A1c-distribution in a healthy population. The Telecom Study. Diabetologia 32: 864–869. 2693166 10.1007/BF00297451

[36] Goldstein DE, Little RR, Lorenz RA, Malone JI, Nathan D, et al (1995) Tests of glycemia in diabetes. Diabetes Care 18: 896–909. 7555528 10.2337/diacare.18.6.896

[37] Mucalo I, Rahelic D, Jovanovski E, Bozikov V, Romic Z, et al (2012) Effect of American ginseng (Panax quinquefolius L.) on glycemic control in type 2 diabetes. Coll Antropol 36: 1435–1440. 23390846

[38] Luo JZ, Luo L (2009) Ginseng on hyperglycemia: effects and mechanisms. Evid Based Complement Alternat Med 6: 423–427. 18955300 10.1093/ecam/nem178 PMC2781779

[39] Yeh GY, Eisenberg DM, Kaptchuk TJ, Phillips RS (2003) Systematic review of herbs and dietary supplements for glycemic control in diabetes. Diabetes Care 26: 1277–1294. 12663610 10.2337/diacare.26.4.1277

[40] Sievenpiper JL, Arnason JT, Vidgen E, Leiter LA, Vuksan V (2004) A systematic quantitative analysis of the literature of the high variability in ginseng (Panax spp.): should ginseng be trusted in diabetes? Diabetes Care 27: 839–840. 10.2337/diacare.27.3.839-a 14988315

