# Supplementary material for: The Effect of Ginseng (The Genus Panax) on Glycemic Control: A Systematic Review and Meta-Analysis of Randomized Controlled Clinical Trials
Source: PLoS One. 2014 Sep 29;9(9):e107391. doi: 10.1371/journal.pone.0107391 (PMC4180277; doi:10.1371/journal.pone.0107391)
Supplement: Figure S1 — Risk of bias assessment for included trials by the Cochrane risk of bias tool. (DOC) [file pone.0107391.s001.doc]

**Figure S1:** Risk of bias graph: review authors' judgements about each risk of bias item presented as percentages across all included studies.
