# Supplementary material for: The Effect of Ginseng (The Genus Panax) on Glycemic Control: A Systematic Review and Meta-Analysis of Randomized Controlled Clinical Trials
Source: PLoS One. 2014 Sep 29;9(9):e107391. doi: 10.1371/journal.pone.0107391 (PMC4180277; doi:10.1371/journal.pone.0107391)
Supplement: Figure S2 — Forest plots of subgroup analyses investigating the effect of ginseng on fasting blood glucose. (DOCX) [file pone.0107391.s002.docx]

**Figure S2**

**Figure S2**. Forest plots of subgroup analyses investigating the effect of ginseng on fasting blood glucose. Data are mean differences (MD) with 95% CI. N represents the number of participants in each subgroup. Between subgroup differences were analyzed using meta-regression, with the residual I^2^ reported as a percent value and significance as a *P*-value, with *P* < 0.05 as significant^*^. All subgroups were pre-specified as *a priori* except for funding (post-hoc).
