# Supplementary material for: The Effect of Ginseng (The Genus Panax) on Glycemic Control: A Systematic Review and Meta-Analysis of Randomized Controlled Clinical Trials
Source: PLoS One. 2014 Sep 29;9(9):e107391. doi: 10.1371/journal.pone.0107391 (PMC4180277; doi:10.1371/journal.pone.0107391)
Supplement: Figure S6 — Funnel plot assessing publication bias and effect of small and/or imprecise study effects in clinical trials. (DOCX) [file pone.0107391.s006.docx]

**Figure S6**

**Figure S6.** Funnel plot assessing publication bias and effect of small and/or imprecise study effects in clinical trials investigating the effects of ginseng on A. Fasting blood glucose, B. Fasting plasma insulin, C. Glycated hemoglobin, and D. Homeostasis model assessment of insulin resistance. The dashed lines represent the pooled effect estimate expressed as a mean difference (MD). The diamonds represent within subgroup MD, and the horizontal lines represent standard errors of the MD.
