# Supplementary material for: The Effect of Ginseng (The Genus Panax) on Glycemic Control: A Systematic Review and Meta-Analysis of Randomized Controlled Clinical Trials
Source: PLoS One. 2014 Sep 29;9(9):e107391. doi: 10.1371/journal.pone.0107391 (PMC4180277; doi:10.1371/journal.pone.0107391)
Supplement: Table S1 — Search strategy for studies assessing the effect of ginseng on glycemic control in randomized controlled trials. (DOCX) [file pone.0107391.s007.docx]

| **Database** | **Search period** | **Search** |
| --- | --- | --- |
| MEDLINE | 1946 to week 1 of July 2013 | 1. exp Panax/ or ginseng.mp. |
|  |  | 2. exp Hemoglobin A, Glycosylated/ or exp Glucose/ or exp Fructosamine/ or fructosamine.mp. or glycemia.mp or exp Hyperglycemia/ or fructosamine*.mp. or diabetes millitus.mp. or exp Insulin/ or insulin.mp. or exp Insulin/ or dysglycemia.mp. or hyperinsulin.mp. or hyperinsulin*.mp. or exp Diabetes Mellitus, Type1/ or type 1 diabetes.mp. or exp Diabetes Mellitus, Type2/ or type 2 diabetes.mp. or gly* albumin.mp. or exp Diabetes, Gestational/ or gestational diabetes.mp. or exp Prediabetic State/ or prediabetes.mp. or HBA1C.mp. |
|  |  | 3. 1 and 2 |
|  |  | 4. Limit 3 to animals |
|  |  | 5. 3 not 4 |
| EMBASE | 1947 to week 27 of 2013 | 1. exp Panax/ or ginseng.mp. |
|  |  | 2. exp Hemoglobin A, Glycosylated/ or exp Glucose/ or exp Fructosamine/ or fructosamine.mp. or glycemia.mp or exp Hyperglycemia/ or fructosamine*.mp. or diabetes millitus.mp. or exp Insulin/ or insulin.mp. or exp Insulin/ or dysglycemia.mp. or hyperinsulin.mp. or hyperinsulin*.mp. or exp Diabetes Mellitus, Type1/ or type 1 diabetes.mp. or exp Diabetes Mellitus, Type2/ or type 2 diabetes.mp. or gly* albumin.mp. or exp Diabetes, Gestational/ or gestational diabetes.mp. or exp Prediabetic State/ or prediabetes.mp. or HBA1C.mp. |
|  |  | 3. 1 and 2 |
|  |  | 4. Limit 3 to animals |
|  |  | 5. 3 not 4 |
| CINAHL | 1985 to 3 July 2013 | (Panax OR panax* OR ginseng* OR ninjin* OR renshen* OR shinseng* OR jen adj shen* OR schinseng* OR Ginsenosides OR Ginsenoside*) AND (“Glucose Tolerance Test” OR OGTT OR Hemoglobin A, Glycosylated OR Hemoglobin A, Glycosylated OR HBA1C OR Fructosamine* OR insulin* OR Glucose OR Hyperglycemia OR Hyperglycaemia OR glycaemia OR hyperinsulin* OR dysglycemia OR “Diabetes Mellitus” OR HOMA* OR glycemia OR “gly* albumin” OR diabetes OR “metabolic syndrome” OR “homeostasis model assessment” OR hyperglycemic OR hyperglycaemic) |
| The Cochrane Library | Through to 3 July 2013 | (Panax OR panax* OR ginseng* OR ninjin* OR renshen* OR shinseng* OR jen adj shen* OR schinseng* OR Ginsenosides OR Ginsenoside*) AND (“Glucose Tolerance Test” OR OGTT OR Hemoglobin A, Glycosylated OR Hemoglobin A, Glycosylated OR HBA1C OR Fructosamine* OR insulin* OR Glucose OR Hyperglycemia OR Hyperglycaemia OR glycaemia OR hyperinsulin* OR dysglycemia OR “Diabetes Mellitus” OR HOMA* OR glycemia OR “gly* albumin” OR diabetes OR “metabolic syndrome” OR “homeostasis model assessment” OR hyperglycemic OR hyperglycaemic) |

**Table S1:** Search strategy for studies assessing the effect of ginseng on glycemic control in randomized controlled trials*

* The initial search for all databases was conducted on October 24, 2012. All databases underwent updated searches on December 12, 2012; March 5, 2013; and July 3, 2013.
