# Supplementary material for: The Effect of Ginseng (The Genus Panax) on Glycemic Control: A Systematic Review and Meta-Analysis of Randomized Controlled Clinical Trials
Source: PLoS One. 2014 Sep 29;9(9):e107391. doi: 10.1371/journal.pone.0107391 (PMC4180277; doi:10.1371/journal.pone.0107391)
Supplement: Table S2 — Study Quality Assessment by the Heyland MQS. (DOCX) [file pone.0107391.s008.docx]

**Table S2:** Study Quality Assessment by the Heyland MQS*

|  | **Methods†** | | | **Sample‡** | | | **Intervention§** | | | **MQS** |
| --- | --- | --- | --- | --- | --- | --- | --- | --- | --- | --- |
| **Study** | Randomization (n/2) | Blinding (n/1) | Analysis (n/2) | Selection (n/1) | Compatibility (n/1) | Follow-up (n/1) | Protocol (n/1) | Co-interventions (n/2) | Crossovers (n/2) | (n/13) |
| **Diabetes** | | | | | | | | | | |
| Chio et al. 1997 [19] | 1 | 0 | 2 | 0 | 1 | 1 | 1 | 2 | 0 | 8 |
| Kim et al. 2011 [21] | 1 | 1 | 0 | 0 | 1 | 0 | 1 | 2 | 0 | 6 |
| Ma et al. 2008 [22] | 1 | 1 | 2 | 0 | 1 | 1 | 0 | 2 | 0 | 8 |
| Reeds et al. 2011 [25] | 2 | 1 | 2 | 0 | 1 | 1 | 1 | 2 | 0 | 10 |
| Sotaniemi et al. 1995 [7] | 1 | 1 | 2 | 1 | 1 | 1 | 0 | 0 | 0 | 7 |
| Vuksan et al. 2000 [29] | 1 | 1 | 0 | 1 | 1 | 0 | 1 | 2 | 0 | 7 |
| Vuksan et al. 2008 [9] | 2 | 1 | 0 | 1 | 1 | 0 | 1 | 2 | 0 | 8 |
| Yoon et al. 2012 [30] | 2 | 1 | 2 | 1 | 1 | 0 | 1 | 2 | 0 | 10 |
| Zhang et al. 2007 [31] | 2 | 0 | 2 | 0 | 1 | 1 | 1 | 1 | 0 | 8 |
| **Non-diabetes** | | | | | | | | | | |
| Dickman et al. 2009 [20] | 1 | 1 | 0 | 1 | 1 | 0 | 1 | 2 | 0 | 7 |
| Park et al. 2012 [23] | 2 | 1 | 0 | 1 | 1 | 0 | 1 | 2 | 0 | 8 |
| Reay et al. (a) 2009 [24] | 1 | 1 | 2 | 1 | 1 | 1 | 1 | 2 | 0 | 10 |
| Reay et al. (b) 2009 [24] | 1 | 1 | 2 | 1 | 1 | 1 | 1 | 2 | 0 | 10 |
| Rhee et al. 2011 [26] | 1 | 1 | 0 | 1 | 1 | 0 | 1 | 2 | 0 | 7 |
| Scaglione et al. 1996 [27] | 2 | 1 | 0 | 1 | 1 | 0 | 1 | 2 | 0 | 8 |
| Shin et al. 2011 [28] | 1 | 1 | 2 | 1 | 1 | 1 | 1 | 2 | 0 | 10 |

^*^ The Heyland MQS assigns a score of 0 or 1 or from 0 to 2 over 9 categories of quality related to study design, sampling procedures, and interventions, for a total of 13 points. Trials that scored ≥ 8 were considered to be of higher quality [17].

^†^ Randomization was scored 2 points for being randomized with the methods described, 1 point for being randomized without the methods described, or 0 points for being neither randomized nor having the methods described. Blinding was scored 1 point for being double-blind or 0 points for “other.” Analysis was scored 2 points for being intention-to-treat; all other types of analyses scored 0 points.

^‡^ Sample selection was scored 1 point for being consecutive eligible or 0 points for being preselected or indeterminate. Sample comparability was scored 1 point for being comparable or 0 points for not being comparable at baseline. Follow-up was scored 1 point for being 100% or 0 points for < 100%.

^§^ Treatment protocol was scored 1 point for being reproducibly described or 0 points for being poorly described. Co-interventions were scored 2 points for being described and equal, 1 point for being described but unequal or indeterminate, or 0 points for not being described. Treatment crossovers (where participants were switched from the control treatment to the experimental treatment) were scored 2 points for being ≥ 10%, 1 point for being < 10%, and 0 points for not being described.
