# Supplementary material for: The Effect of Ginseng (The Genus Panax) on Glycemic Control: A Systematic Review and Meta-Analysis of Randomized Controlled Clinical Trials
Source: PLoS One. 2014 Sep 29;9(9):e107391. doi: 10.1371/journal.pone.0107391 (PMC4180277; doi:10.1371/journal.pone.0107391)
Supplement: Table S3 — Continuous meta-regression analysis for the effect of ginseng on glycemic parameters. (DOCX) [file pone.0107391.s009.docx]

**Table S3:** Continuous meta-regression analysis for the effect of ginseng on glycemic parameters

| **Subgroup** | | **Range** | **No. of trials** | **N** | **β [95% CI]** | **Residual I^2^** | ***P*-value** |
| --- | --- | --- | --- | --- | --- | --- | --- |
| **FBG** | | | | | | | |
|  | Baseline (mmol/L) | 4.5 – 13.2 | 16 | 770 | -0.26 [-0.40, -0.13] | 70.4% | 0.001^*^ |
|  | Duration (weeks) | 4 – 24 | 16 | 770 | -0.02 [-0.11, 0.07] | 89.7% | 0.66 |
| **FPI** | | | | | | | |
|  | Baseline (pmol/L) | 41 – 149 | 10 | 347 | 0.03 [-0.33, 0.40] | 29.3% | 0.85 |
|  | Duration (weeks) | 4 – 12 | 10 | 347 | -1.19 [-2.80, 0.42] | 7.1% | 0.13 |
| **HbA1c** | | | | | | | |
|  | Baseline (%) | 5.4 – 9.6 | 9 | 264 | -0.11 [-0.28, 0.06] | 51.7% | 0.18 |
|  | Duration (weeks) | 4 – 24 | 9 | 264 | -0.009 [-0.05, 0.03] | 52.3% | 0.61 |
| **HOMA-IR** | | | | | | | |
|  | Baseline | 1.04 – 7.4 | 7 | 303 | -0.25 [-0.73, 0.23] | 83.6% | 0.24 |
|  | Duration (weeks) | 4 – 12 | 7 | 303 | 0.13 [-0.14, 0.40] | 81.5% | 0.28 |

Abbreviations: FBG: Fasting blood glucose; FPI: Fasting plasma insulin; HbA1c: Glycated haemoglobin; HOMA-IR: Homeostasis model assessment of insulin resistance. Changes in mean-difference between ginseng and control intervention per unit change in each predictor using continuous meta-regressions. A positive β-coefficient implies an increase in FBG, FPI, HbA1c, or HOMA-IR response with ginseng supplementation relative to control; and a negative β-coefficient implies a decrease in FBG, FPI, HbA1c, or HOMA-IR response with ginseng supplementation relative to control. ^*^ Indicates significance at *P* > 0.05.
